# Exposure to Air Pollution Exacerbates Inflammation in Rats with Preexisting COPD

**DOI:** 10.1155/2020/4260204

**Published:** 2020-05-08

**Authors:** Jing Wang, Ya Li, Peng Zhao, Yange Tian, Xuefang Liu, Huihui He, Rui Jia, Brian G. Oliver, Jiansheng Li

**Affiliations:** ^1^Henan Key Laboratory of Chinese Medicine for Respiratory Disease, Henan University of Chinese Medicine, Zhengzhou 450046, China; ^2^Co-construction Collaborative Innovation Center for Chinese Medicine and Respiratory Diseases by Henan & Education Ministry of P.R. China, Zhengzhou 450046, China; ^3^Academy of Chinese Medicine, Henan University of Chinese Medicine, Zhengzhou, Henan 450046, China; ^4^Institute of Respiratory Disease and Centre Laboratory, The First Affiliated Hospital, Henan University of Chinese Medicine, Zhengzhou 450000, China; ^5^School of Life Sciences, Faculty of Science, University of Technology Sydney, Sydney, New South Wales 2007, Australia; ^6^Woolcock Institute of Medical Research, Respiratory Cellular and Molecular Biology, The University of Sydney, New South Wales 2037, Australia

## Abstract

Particulate matter with an aerodynamic diameter equal or less than 2.5 micrometers (PM2.5) is associated with the development of chronic obstructive pulmonary disease (COPD). The mechanisms by which PM2.5 accelerates disease progression in COPD are poorly understood. In this study, we aimed to investigate the effect of PM2.5 on lung injury in rats with hallmark features of COPD. Cardinal features of human COPD were induced in a rat model by repeated cigarette smoke inhalation and bacterial infection for 8 weeks. Then, from week 9 to week 16, some of these rats with COPD were subjected to real-time concentrated atmospheric PM2.5. Lung function, pathology, inflammatory cytokines, oxidative stress, and mucus and collagen production were measured. As expected, the COPD rats had developed emphysema, inflammation, and deterioration in lung function. PM2.5 exposure resulted in greater lung function decline and histopathological changes, as reflected by increased Mucin (MUC) 5ac, MUC5b, Collagen I, Collagen III, and the profibrotic cytokine *α*-smooth muscle-actin (SMA), transforming growth factor- (TGF-) *β*1 in lung tissues. PM2.5 also aggravated inflammation, increasing neutrophils and eosinophils in bronchoalveolar lavage fluid (BALF) and cytokines including Interleukin- (IL-) 1*β*, granulocyte-macrophage colony-stimulating factor (GM-CSF), and IL-4. The likely mechanism is through oxidative stress as antioxidants levels were decreased, whereas oxidants were increased, indicating a detrimental shift in the oxidant-antioxidant balance. Altogether, these results suggest that PM2.5 exposure could promote the development of COPD by impairing lung function and exacerbating pulmonary injury, and the potential mechanisms are related to inflammatory response and oxidative stress.

## 1. Introduction

Chronic obstructive pulmonary disease (COPD) is an international health problem with a rising prevalence and mortality and is estimated to be the third commonest cause of death and the fifth leading cause of disability by 2020 [1, 2]. It is characterized by persistent airflow limitation and chronic airway inflammation typically caused and worsened by inhalation of noxious gases or particles [3]. Among the major causes of COPD, the role of cigarette smoking is widely recognized. However, there is current evidence that a substantial proportion of COPD cases are never smokers, especially among women and residents of developing countries [4–6]. According to Global Burden of Disease (GBD) study, 19.3% of disability-adjusted life-years (DALYs) in COPD were attributable to particulate matter (PM) exposure [7]. Numerous studies have indicated the association between increased hospitalization, morbidity, and mortality of COPD [8, 9], as well as aggravated respiratory function and symptoms [10–12], and the short-term exposure to ambient particulate air pollution including PM2.5. Ambient PM2.5 has been recognized as a major detrimental risk factor for the development, progression, and exacerbation of COPD.

However, our understanding of PM2.5 for the development of COPD is still incomplete and only a few studies have described the related mechanisms in greater details. PM2.5 is a complex mixture of solid and liquid elements suspended in the atmosphere, being generated from industry, coal combustion, biomass fuel for cooking, and traffic pollution [13]. Its toxicity is determined by size, components, origin, and ability to produce reactive oxygen. PM2.5 has small diameters but large surface areas and therefore capable of carrying several various toxic components including water-soluble ions, transition metals, reactive gases, and polycyclic aromatic hydrocarbons (PAHs). Due to its small size and easy transportation, PM2.5 can be inhaled deep into human airway and deposited in lung tissues, especially alveolar regions, causing local damage in the lungs [14]. There have been evidences that PM2.5 has a harmful impact on lung function and alveolar structure, and related mechanisms are focused on inflammatory response, oxidative stress, immune disorders, and genetic alterations [15–18]. In our previous studies, short-term exposure of healthy rats to high-concentration PM2.5 led to a burst of inflammation and oxidative stress in the lungs, as well as the ciliary dysfunction in the upper airway.

It is well known that in COPD progressive airflow limitation generally results from two major pathological processes, small airway remodeling and narrowing resulting from loss of alveolar attachments as a result of emphysema [19]. Airway wall thickening is caused by epithelial cell hyperplasia, goblet cell metaplasia, and peribronchial fibrosis and is characteristic of the pathology seen in the lungs of COPD patients [20]. Longstanding inflammation is widely considered to play a critical role in airway remodeling [21]. Mucus hypersecretion may contribute to airway occlusion as mucus occupies the airway lumen and tends to be retained because of ciliary dysfunction [22], while several matrix metalloproteinases (MMPs) that degrade extracellular matrix (ECM) are actively involved in the destruction of lung parenchyma that leads to emphysema [23]. Degradation of ECM results in both the liberation of matikines and latent ECM-bound growth factors such as transforming growth factor- (TGF-) *β*, resulting in lung fibrosis [24, 25].

Here, based on the successful replication of COPD rat model, we used a whole-body PM2.5 exposure system to explore the impact of exposure to PM2.5 on lung injury of rats with preexisting COPD. Changes in lung function, pulmonary histopathology, inflammatory cytokines, oxidative stress, mucus secretion, proteinases/antiproteinases, and the level of airway remodeling were assessed.

## 2. Material and Methods

### 2.1. Animals

Twenty-eight male Sprague-Dawley (SD) rats (200 ± 20 g) were obtained from the Experimental Animal Center of Shandong Province (SCXK (Lu) 20140007, Jinan, China). All rats were fed a normal diet and housed in cages without beddings but under standard conditions of humidity (50 ± 10%), temperature (25 ± 2°C), and light (12 h light/12 h dark cycle).

### 2.2. COPD Rat Model Establishment and Whole-Body PM2.5 Exposure

These 28 rats were randomly divided into control, PM2.5, COPD, and PM2.5+COPD groups (7 rats per group). The COPD rat model was prepared by using repeated cigarette smoke inhalation and bacterial infection as described previously [26]. Briefly, after equilibrating for 7 days, rats in the COPD and PM2.5+COPD groups were exposed to cigarette smoke (Hongqiqu® Filter Cigarette, Henan Tobacco Industry, Zhengzhou, China; each cigarette contained 1.0 mg nicotine, 11 mg CO, and 10 mg tar oil) in a self-made exposure chamber (length: 1500 cm, width: 800 cm, and height: 2500 cm), 30 minutes each time (smoke concentration: 3000 ± 500 ppm), twice daily, with 3-hour smoke-free intervals, 6 days per week. Additionally, Klebsiella pneumonia suspension (strain ID: 46114, National Center for Medical Culture Collection, Beijing, China; 0.1 mL, 6 × 10^8^ colony forming units/mL) was slowly dropped into two nasal cavities of rats, once every 5 days, continuously for 8 weeks. The successful establishment of the COPD rat model was decided by decreased pulmonary function and pathological changes in lung tissues such as pulmonary and airway inflammation, alveolar wall thickening, alveolar cavity enlargement.

**Figure 1:**
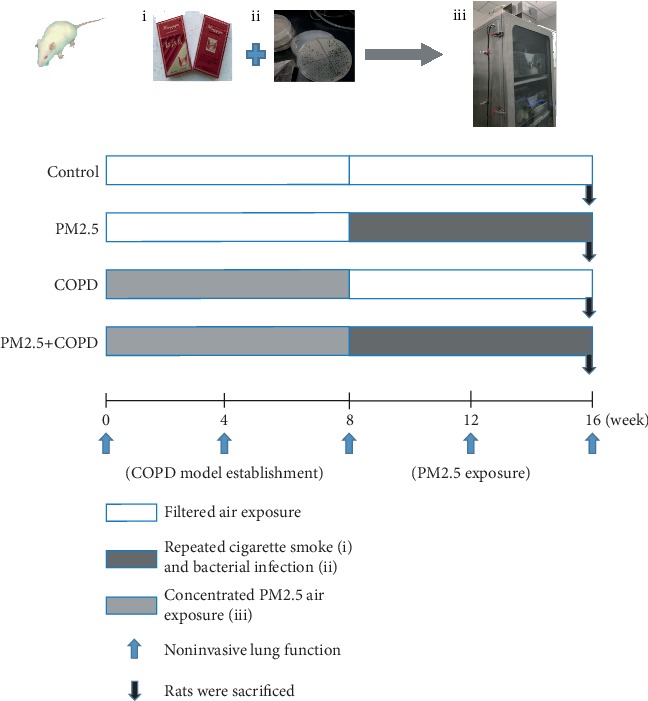
Experimental protocol. SD rats were divided into control, PM2.5, COPD, and PM2.5+COPD groups. During weeks 0~8, for the COPD and PM2.5+COPD groups, the COPD rat model was prepared by using repeated cigarette smoke inhalation and bacterial infection. During weeks 8~16, rats in the PM2.5 and PM2.5+COPD groups were exposed to concentrated PM2.5 atmosphere in the whole-body exposure chamber, 4 hours per day. Rats in the control group were exposed to filtered air all the time. At weeks 0, 4, 8, 12, and 16, the noninvasive lung function (TV, PEF, and EF50) were measured. All rats were sacrificed within 24 h of the last PM2.5 exposure. And lung function, pulmonary histopathology, inflammatory cytokines, oxidative stress, mucus secretion, and proteinases/antiproteinases were detected. Control: healthy rats; PM2.5: particulate matter 2.5 treatment alone rats; COPD: chronic obstructive pulmonary disease rats; PM2.5+COPD: COPD rats exposed to prolonged PM2.5 for 8 weeks. TV: tidal volume; PEF: peak expiratory flow; EF50: expiratory flow 50%.

Then, in the following 8 weeks, rats in the control and COPD groups were exposed to filtered air (PM2.5 concentration < 10 *μ*g/m^3^), while rats in the PM2.5 and PM2.5+COPD groups were exposed to concentrated PM2.5 atmosphere in the whole-body exposure chamber (1.2 m^3^). The exposure protocol was 4 hours per day (8:00 am~12:00 am), 6 days per week from 30 November 2018 to 24 January 2019. All rats were anaesthetized with 3% pentobarbital sodium (0.25 mL/100 g body weight) within 24 h of the last PM2.5 exposure for collection of blood, BALF, and lung tissues (see the flow chart of the experiment design in Figure 1).

### 2.3. The Parameters Monitored in Exposure Equipment

The concentrated PM2.5 was generated using a PM2.5 concentration enrichment system (Beijing Huironghe Technology Co., Ltd, Beijing, China) located at the new campus of Henan University of Chinese Medicine Zhengzhou, China, surrounded by a complex of residences, schools, traffic, and many small-scale factories. This system could make PM2.5 6~10-fold concentrated compared with the ambient.

The real-time concentrations of PM2.5 in the chamber were monitored using a PM2.5 concentration monitor (TSI instrument, MN, USA), and the distribution of particle size was measured by an Aerodynamic Particle Size Analyzer (TSI instrument, MN, USA). Humidities and temperatures were also monitored continuously to keep a standard condition of humidity (40~60%) and temperature (21~27°C) in the chamber. During the exposure period, we collected PM2.5 on the polypropylene filter (aperture: 0.8 *μ*m, Beijing, China) that is placed at the outlet of the chamber, and then, PM2.5 were extracted for component analysis. Briefly, all filters were cut into small pieces and put in a 50 mL centrifuge tube. After ultrasonic washing and shaking for three times repeatedly, the extracts were passed sequentially through eight pieces of sterile gauzes and dried by lyophilization [27]. Then, 1 mg of the PM2.5 powder was used for component analysis. Ions were analyzed by Ion Chromatography (IC) using Thermo Fisher Scientific Dionex ICS-2100. Microelements were analyzed by Inductively Coupled Plasma Mass Spectrometry (ICP-MS) using Thermo Fisher Scientific iCAPQ; PAHs were analyzed by Gas Chromatography and Mass Spectra (GC-MS) using Thermo Fisher Scientific Trace ISQ.

### 2.4. Lung Histology

After 10% formalin fixing and paraffin embedding, the lung tissue samples were sectioned into 4 *μ*m thick slides and stained with hematoxylin and eosin (HE) for histology and morphometric analysis with an LEICA-DM6000B microscope.

Total lung injury score (LIS) was defined by the average score of alveolar, bronchial, and vascular injury, characterized by inflammatory infiltration, alveolar wall rupture and fusion, based on Smith's: 0 = no detectable injury, 1 = less than 25% injury, 2 = 25%~50% injury, 3 = 50%~75% injury, and 4 = more than 75% injury [28]. Then, quantification of emphysematous lesions was determined by mean linear intercepts (MLI) and mean alveolar area (MAA) according to Koike's [29]. Briefly, the MLI was obtained from the results of total length (L) of each line of grid divided by the number of alveolar intercepts (NAI). The MAA was defined as the results of airspace surfaces (S) divided by the number of alveoli (NA). All analyses were conducted by two trained pathologists blinded to the study protocol.

### 2.5. Pulmonary Function

Tidal volume (TV), peak expiratory flow (PEF), and expiratory flow at 50% tidal volume (EF50) were measured by an unrestrained whole body plethysmograph (Buxco Inc., Wilmington, NC, USA) at weeks 0, 4, 8, 12, and 16. By the end of week 16, forced expiratory vital capacity (FVC), forced expiratory volume in 0.3 s (FEV0.3), and FEV0.3/FVC were measured after rats were anaesthetized and inserted with a tracheal cannula by using a computer-controlled pulmonary function test system (Buxco Inc., DSI, St. Paul, MN, USA).

### 2.6. Collection of Bronchoalveolar Lavage Fluid (BALF) and Cell Classification

After rats were sacrificed, the left lungs were lavaged twice with 3 mL of phosphate-buffered solution (PBS) through tracheal intubation and up to 50% recovery was obtained. 10 *μ*L of the recovered lavage fluid was used for total cell counts using cell-count boards. The BALF supernatant and sediment were then obtained by centrifugation at 1200 rpm for 10 minutes in 4°C. Numbers of eosinophils, neutrophils, and macrophages were determined in a total of 200 cells using HE staining under microscope. All analyses were conducted by two trained pathologists blinded to the study protocol.

### 2.7. Enzyme-Linked Immunosorbent Assay

Levels of Interleukin- (IL-) 1*β*, IL-4, granulocyte-macrophage colony-stimulating factor (GM-CSF), matrix metalloproteinase 9 (MMP-9), and matrix metalloproteinase 12 (MMP-12) and tissue inhibitor of metalloproteinases (TIMP-1) were measured using individual ELISA kits (Boster/Elabscience Technology Co., Wuhan, China) under the manufacturer's instructions.

### 2.8. Western Blotting

Total protein was extracted from lung tissues using cold RIPA buffer containing protease and phosphatase inhibitors (Solarbio, Beijing, China). The protein expressions of nuclear factor erythroid 2-related factor 2 (Nrf-2) and heme oxygenase-1 (HO-1) in lung tissues were measured using Western blot. Anti-Nrf-2 Ab (1 : 1000, LiankeBio, Hangzhou, China), anti-HO-1 Ab (1 : 500, GeneTex, USA), and anti-GAPDH Ab (1 : 5000, GeneTex, USA) were used. ChemiDoc™ MP (Bio-Rad, Hercules, CA, USA) imaging system was used to record signals. ImageJ was used to quantify the intensity of the protein band, which was normalized to GAPDH in the analysis.

### 2.9. Immunohistochemistry

After heat fixed, deparaffinized, and rehydrated through graded alcohols to distilled water, lung tissue paraffin sections (4 *μ*m) were blocked with 5% bull serum albumin and incubated with the primary antibodies against Mucin (MUC) 5ac, MUC5b, transforming growth factor- (TGF-) *β*1, *α*-smooth muscle-actin (SMA) (Bioss, Beijing, China), and Collagen I, Collagen III (Proteintech, Wuhan, China) overnight at 4°C. Subsequently, the sections were incubated with goat anti-rabbit IgG at 37°C for 30 minutes and counterstained by hematoxylin according to the manufacturers' instructions (Boster, Wuhan, China). Finally, images were captured and analyzed using the Image-ProPlus 6.0 software (Media Cybernetics, MD, USA).

### 2.10. Measurement of MDA and T-SOD in Serum and BALF

Activity of malondialdehyde (MDA) and total superoxide dismutase (T-SOD) in serum and BALF was measured by hydroxylamine and thiobarbituric acid methods, respectively, according to the manufacturer's protocol (Nanjing Jiancheng Co., Jiangsu, China).

### 2.11. Statistical Analysis

All statistical analyses were performed by SPSS software, version 23.0, for Windows (IBM, Armonk, NY, USA). Data were presented as mean ± standard deviation (SD). One-way analysis of variance (ANOVA) was used to determine statistical differences between the groups. A *p* value of <0.05 was considered statistically significant.

## 3. Results

### 3.1. PM2.5 Concentration

The maximum and minimum concentrations of PM2.5 in the chamber were 2227.64 *μ*g/m^3^ and 205.12 *μ*g/m^3^, respectively. The average concentration for 8 weeks of exposure was 739.97 *μ*g/m^3^. Figure S1 (Supplementary Materials) shows the daily atmospheric concentration of PM2.5 after being concentrated during the exposure period.

### 3.2. PM2.5 Exposure Aggravated Lung Histological Injury in COPD Rats

**Figure 2:**
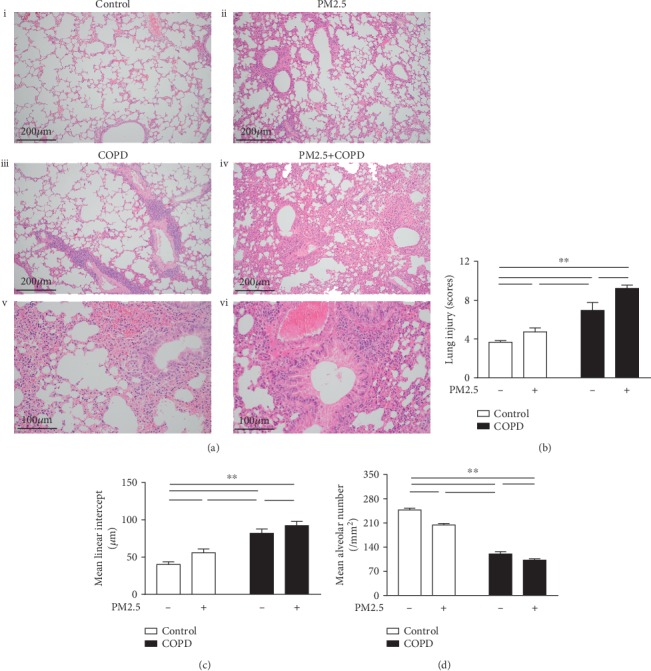
The effect of PM2.5 on lung histological injury in rats with COPD. (a) Lung tissue HE staining of different groups of rats. (i) Control, an intact structure of pulmonary alveoli and airway (magnification, ×100); (ii) PM2.5, mild infiltration of inflammatory cells and thickening of airway wall (magnification, ×100); (iii) COPD, alveolar cavity enlargement and vascular wall thickening (magnification, ×100); (iv) PM2.5+COPD, chronic obstructive bronchiolitis with thickening of the airway wall and infiltration with inflammatory cells (magnification, ×100); (v) COPD, airway wall thickening and inflammatory cell infiltration (magnification, ×200); (vi) PM2.5+COPD, increased thickening of airway wall and infiltration of inflammatory cells compared to COPD (magnification, ×200). (b–d) Quantitative analysis of lung injury, that is, the level of (b) LIS, (c) MLI, and (d) MAN of the lungs in each group. LIS: lung injury scores; MLI: mean linear intercept; MAN: mean alveolar number. The data are expressed as the means ± SD (*n* = 6~7). ^∗∗^*p* < 0.01, ^∗^*p* < 0.05.

As shown in Figure 2, the structure of pulmonary alveoli and airway was fully intact in control rats. In contrast, rats in the COPD group showed alterations in both alveolar and tracheal areas, including inflammatory cell infiltration, alveolar wall thickening, alveolar cavity enlargement, and small conducting airway thickening. In comparison, PM2.5 alone rats were less severe. PM2.5 exposure further increased infiltration of inflammatory cells and thickening of alveolar and airway wall in COPD rats, but without amplifying alveolar cavity.

We then used LIS, MLI, and MAN for quantification of this lung injury and found both LIS and MLI increased, whereas MAN decreased in all 3 treated groups in comparison to the control. Meanwhile, a further increase in LIS and MLI and a further decrease in MAN were observed in the rats with combined exposure.

### 3.3. PM2.5 Exposure Promoted Airway Remodeling in COPD Rats

**Figure 3:**
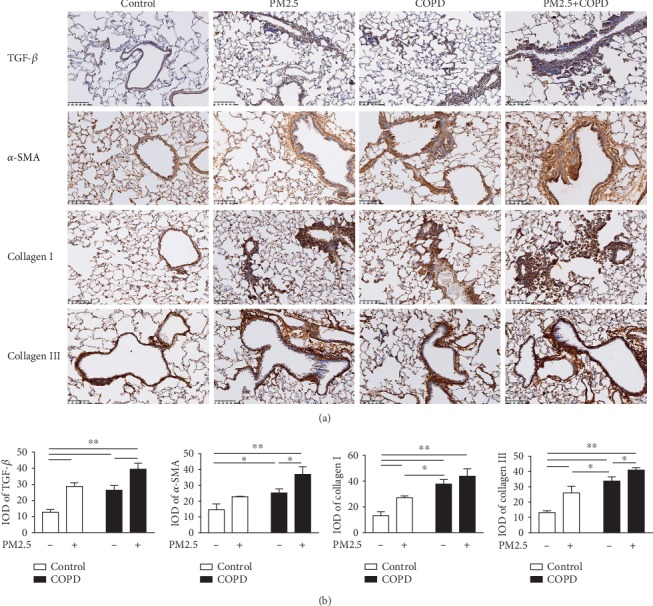
The effect of PM2.5 on airway remodeling in rats with COPD. (a) Immunohistochemical staining of TGF-*β*, *α*-SMA, Collagen I, and Collagen III of lung sections in each group (magnification, ×200). (b) Quantitative analysis of TGF-*β*, *α*-SMA, Collagen I, and Collagen III using Image-ProPlus 6.0 software. TGF-*β*1: transforming growth factor-beta 1; *α*-SMA: *α*-smooth muscle-actin; IOD: integral optical density. The data are expressed as the means ± SD (*n* = 6). ^∗∗^*p* < 0.01, ^∗^*p* < 0.05.

Airway remodeling occurs in COPD and is positively correlated to COPD severity. The major contributor to this is increased ECM proteins [30]. TGF-*β*1 is a major profibrotic cytokine which induces ECM deposition [25]. As shown in Figure 3, a significant upregulation in the protein expression of TGF-*β*1 was observed in lung tissues of PM2.5 and COPD rats and further elevated in combined treated rats. Similarly, Collagen I and Collagen III, other predominant ECM proteins [31], were markedly increased in these treatment groups, and the combined treatment was higher than PM2.5 or COPD alone. Airway smooth muscle (ASM) hypertrophy is another hallmark of airway remodeling [25]. We next detected the lung protein expression of ASM marker *α*-SMA and found that it significantly increased in all 3 treatment groups, with the highest in the PM2.5+COPD group.

### 3.4. PM2.5 Exposure Impaired Pulmonary Function in COPD Rats

**Figure 4:**
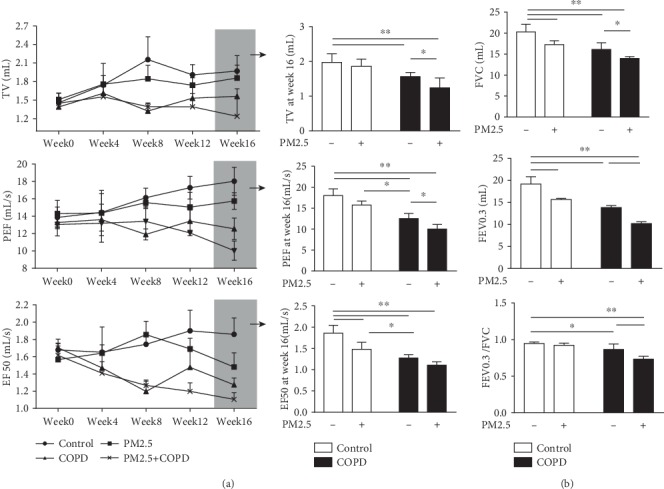
The effect of PM2.5 on pulmonary function in rats with COPD. (a) The change of noninvasive lung function parameters TV, PEF, and EF50 of rats in each group from week 0 to week 16, as well as at week 16. (b) The change of invasive lung function parameters FVC, FEV0.3, and FEV0.3/FVC of rats in each group at week 16. TV: tidal volume; PEF: peak expiratory flow; EF50: expiratory flow 50%; FVC: forced vital capacity; FEV0.3: forced expiratory volume at 0.3 s; FEV0.3/FVC: forced expiratory volume at 0.3 s/forced vital capacity. The data are expressed as the means ± SD (*n* = 7). ^∗∗^*p* < 0.01, ^∗^*p* < 0.05.

Pulmonary function is an important indicator for respiratory disease development. As shown in Figure 4, all 3 treated groups had significantly decreased TV, PEF, and EF50 in comparison to control. In COPD rats, these three noninvasive parameters declined with time and were stable from week 8 onwards. In rats with combined exposure, a further decrease in TV, PEF, and EF50 occurred after PM2.5 exposure. Similarly, the invasive lung function parameters FVC, FEV0.3, and FEV0.3/FVC were also reduced in COPD rats and further decreased in the combined treatments groups.

### 3.5. PM2.5 Exposure Enhanced Inflammatory Response in COPD Rats

**Figure 5:**
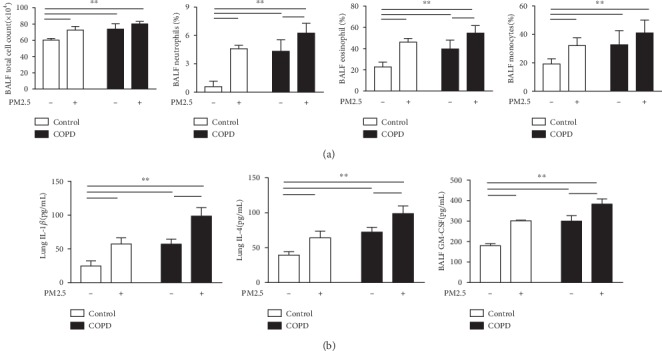
The effect of PM2.5 on inflammatory response in rats with COPD. (a) The total cell count and percentage of neutrophils, eosinophils, and macrophages in the BALF of rats in each group. (b) Level of IL-1*β* and IL-4 in the lung and GM-CSF in the BALF of rats in each group. IL-1*β*: Interleukin-1*β*; IL-4: Interleukin-4; GM-CSF: granulocyte-macrophage colony-stimulating factor. The data are expressed as the means ± SD (*n* = 7). ^∗∗^*p* < 0.01, ^∗^*p* < 0.05.

COPD is associated with chronic lung inflammation. As shown in Figure 5(a), the total amount of cells in BALF and the percentage of eosinophils, neutrophils, and macrophages in the PM2.5, COPD, and PM2.5+COPD groups were higher than those in the control group. And compared to the COPD group, the percent of neutrophils and eosinophils in the PM2.5+COPD group was significantly increased.

As shown in Figure 5(b), some inflammatory cytokines were detected. Levels of IL-1*β*, GM-CSF, and IL-4 were markedly upregulated in the three treatment groups compared to the control rats. PM2.5 exposure, moreover, markedly enhanced these three proinflammatory cytokines in COPD rats.

### 3.6. PM2.5 Exposure Worsened Oxidative Stress in COPD Rats

**Figure 6:**
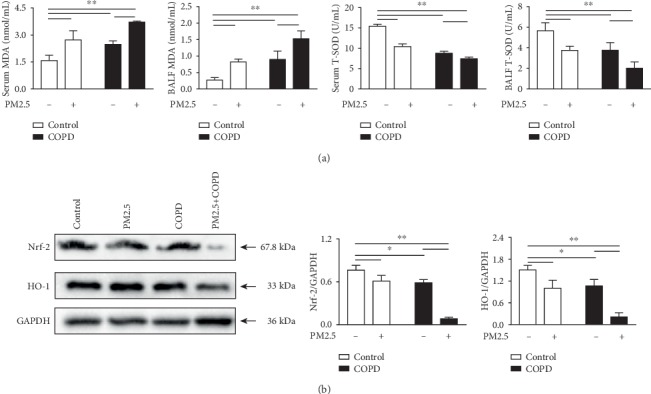
The effect of PM2.5 on oxidative stress in rats with COPD. (a) The levels of MDA and T-SOD in the serum and BALF of rats in each group. (b) Protein expression levels of Nrf-2 and HO-1 in the lung measured by Western blotting. MDA: malondialdehyde; T-SOD: total superoxide dismutase; Nrf-2: nuclear factor erythroid 2-related factor 2; HO-1: heme oxygenase-1. The data are expressed as the means ± SD (*n* = 3~7). ^∗∗^*p* < 0.01, ^∗^*p* < 0.05.

Oxidative stress is a critical driving mechanism in COPD [32]. To investigate the effect of PM2.5 on oxidative stress in COPD rats, the expressions of antioxidants and oxidants in serum and BALF were measured. As shown in Figures 6(a), T-SOD activity was markedly decreased in both serum and BALF of PM2.5, COPD, and PM2.5+COPD rats, and this decrease in the PM2.5+COPD group is greater than that in the COPD group. On the other hand, MDA, a biomarker of lipid peroxidation, was markedly increased by all treatments and further increased in the PM2.5+COPD group.

Nrf-2 is a redox-sensitive transcription factor inducing antioxidant expression and negatively associated with the severity of COPD [33, 34]. We evaluated the protein levels of Nrf2 and its major downstream factor HO-1 in the lungs of rats by Western blot. As shown in Figure 6(b), PM2.5 exposure clearly decreased the levels of Nrf2 and HO-1 protein in COPD rats.

### 3.7. PM2.5 Exposure Increased Mucus Secretion in COPD Rat

**Figure 7:**
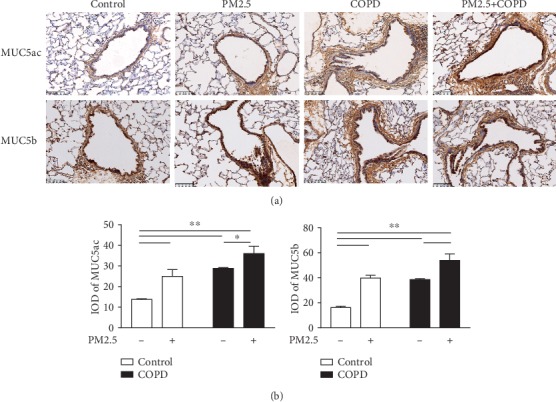
The effect of PM2.5 exposure on mucus hypersecretion in rats with COPD. (a) Immunohistochemical staining of MUC5ac and MUC5b in the lung sections of each group (magnification, ×200). (b) Quantitative analysis of MUC5ac and MUC5b using Image-ProPlus 6.0 software. MUC5ac: Mucin5ac; MUC5b: Mucin5b; IOD: integral optical density. The data are expressed as the means ± SD (*n* = 6). ^∗∗^*p* < 0.01, ^∗^*p* < 0.05.

In COPD, mucus hypersecretion is not only one of the most frequent symptoms but also a critical pathological factor. As shown in Figure 7, MUC5ac and MUC5b, the predominant mucins that contribute to the viscoelastic properties of mucus [22], both were increased in the PM2.5 and COPD rats and further increased in the combined treatment groups. This data indicated that PM2.5 exposure enhanced mucus hypersecretion in COPD rats.

### 3.8. PM2.5 Exposure Elevated Protease-Antiprotease Imbalance in COPD Rats

**Figure 8:**
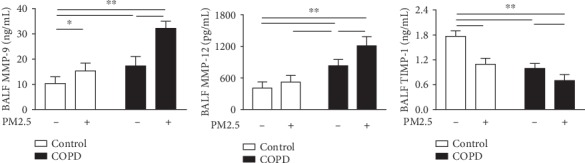
The effect of PM2.5 on protease-antiprotease imbalance in rats with COPD. Level of MMP-9, MMP-12, and TIMP-1 in the BALF of rats in each group. MMP-9: matrix mettaloproteinase-9; TIMP-1: tissue inhibitors of metalloproteases-1. The data are expressed as the means ± SD (*n* = 7). ^∗∗^*p* < 0.01, ^∗^*p* < 0.05.

Another plausible mechanism for the development of emphysema is the overproduction of proteases such as MMPs relative to their inhibitors TIMPs [23]. In this study, we measured MMP-9, MMP-12, and TIMP-1. As shown in Figure 8, the PM2.5 and COPD groups had elevated MMP-9 and MMP-12 and decreased in TIMP-1. Moreover, MMP-9 and MMP-12 in combined treatment were further increased and TIMP-1 further decreased relative to the individual treatment groups.

## 4. Discussion

Our results demonstrated that PM2.5 exposure significantly impaired lung function and histology in COPD rats. Moreover, inflammation, oxidative stress, mucus hypersecretion, and protease-antiprotease imbalance were worsened in COPD rats after PM2.5 exposure. This is the first study to evaluate the effect of PM2.5 on preexisting COPD in multiple aspects using a whole-body PM2.5 exposure system, which mimic the clinical situation in many countries with episodic increases in PM2.5 pollution.

WHO data shows that around 91% of the world's population live in places where air quality levels exceed WHO guideline limits, with developing low-income and middle-income countries suffering from the highest exposures, both indoors and outdoors [35]. To decrease ambient PM2.5 levels, governments worldwide have introduced air quality guidelines. According to WHO ambient air quality guidelines, the suggested PM2.5 concentration limit is 25 *μ*g/m^3^ for the 24-hour mean, and it is 75 *μ*g/m^3^ in China. However, levels are frequently exceeded. In China, rapid economic development and urbanization have a real cost to the environment. Air pollution has posed the fourth highest risk factor contributing to deaths and DALYs in China [36]. PM2.5 levels in 56.2% of 338 Chinese major cities in 2018 exceeded the concentration limit of China [37]. Located in central China, Zhengzhou has a temperate continental monsoon climate with cold-dry winter and ranked in the top ten of the most air polluted cities in China [38]. In winter, notably, its PM2.5 emission usually showed an upward trend owing to the coal combustion for space heating purposes during its 4-month long heating periods [39, 40]. For this reason, a period from November 30, 2018, to January 24, 2019, was chosen for PM2.5 exposure in this study. The data showed that the average daily PM2.5 concentration was 739.97 *μ*g/m^3^ in the chamber, which was far more than the limit of both WHO and China, the concentration enrichment equipment realizing a short-term PM2.5 infection. In addition, this level was close to that in our previous study (626.9 *μ*g/m^3^), which was also performed in a winter heating period from 15 December to 28 December 2017. So it is reasonable to suppose that the average PM2.5 concentration monitored in the same place and time period is repeatable by using this equipment.

Our COPD rat model is generated by a combination of cigarette smoke exposure and repeated bacterial infections and recapitulates many features of human COPD. Importantly, in this model, the impaired pulmonary function and pathological changes in the lungs were irreversible over the 32-week observation period [26]. In the present study, we examined the rats' pulmonary function and lung tissue pathology to verify the replication of the COPD model. The lung function (TV, PEF, and EF50) in the COPD group declined with time from week 1 to week 8, being stable over the 16-week experiment period. By the end of week 16, both noninvasive (TV, PEF, and EF50) and invasive (FVC, FEV0.3, and FEV0.3/FVC) lung function parameters in the COPD group were significantly lower in comparison to those in the control, indicating an irreversible airflow limitation in COPD rats. The pathological results suggested that rats in the COPD group developed more lung tissue injury than controls, including inflammatory cell infiltration, alveolar wall thickening, alveolar cavity enlargement, and small conducting airway thickening.

Based on the successful establishment of the COPD rat model, we used a whole-body PM2.5 exposure system to replicate COPD rats exposed to concentrated PM2.5 atmosphere. In the chamber, more than 90% particles were less than 2.5 *μ*m (see Table S1 in Supplementary Materials). After 8 weeks of exposure, an exacerbating effect of PM2.5 exposure on functional and histological lung damage was readily observed in COPD rats. These findings highlighted the potential use of such method to contribute to animal studies on the chronic hazards of air pollutants to human health. And we hypothesize that this exposure method is more realistic than other previous methods, such as intratracheal instillation in the form of PM2.5 suspension liquid with a stable PM2.5 concentration or ultrasonic nebulization in the form of PM2.5 aerosols [17, 18, 40, 41]. However, it is regrettable that the levels of other gas pollutants including ozone, SO_2_, and NO_2_, which have also been demonstrated to contribute to lung injury and COPD development, were ignored to be measured in this study [42]. But according to Chu et al. and Zhou et al.'s studies, in which the same PM2.5 exposure equipment was used, concentrations of CO, O_3_, NO_2_, and SO_2_ were monitored both in filtered air and concentrated PM2.5 air chambers and no significant differences were found during the exposure [43, 44].

PM2.5 consists of a complex mixture of toxic constituents including free radicals, transition metals, organic chemicals, and microbial components that play critical roles in biological pathogenicity [14]. Metals attached to the surface of PM2.5 trigger a series of catalytic reactions and free radical formation, leading to lipid peroxidation in the cell membrane and inflammation [45]. PM2.5-bound PAHs can cause oxidative damage by activating cytochrome P450 family enzymes, which may upregulate unstable intermediary metabolites and impair human bronchial epithelium [46]. In the present study with repeated measurements, abundant NO3^−^, NH4^+^, SO4^2-^, Fe, Pb, and K, as well as kinds of PAHs, were detected in these collected PM2.5 samples (see Table S2 in Supplementary Materials). The particles breach of mucociliary barrier, reach distal airways, and deposit in alveolar regions [47], thereby eliciting oxidative stress with broken DNA strands, protein carbonylation, lipid peroxidation, and impaired antioxidant system [48].

COPD sufferers may be particularly susceptible to the adverse effects of particle pollutants due to the preexisting impaired lung function, chronic airway inflammation, and ineffective clearance ability [49]. After PM2.5 exposure, an obvious augment in oxidant-antioxidant imbalance was observed in COPD rats, as reflected by increased MDA level while decreased T-SOD activation in the PM2.5+COPD group compared to those in the COPD group. Besides, most antioxidants are regulated by the transcription factor Nrf2, which is negatively associated with the severity of COPD. In this context, the expression of Nrf2 protein, as well as its downstream factor HO-1, showed a downward trend in the lungs of PM2.5-exposed COPD rats.

This PM2.5-increased oxidative stress may stimulate airway epithelial cells and surface macrophages to release more inflammatory mediators such as IL-1*β*, GM-CSF, and IL-4, resulting in an enhancement in chronic inflammation in COPD rats exposed to PM2.5 inhalation. These cytokines, furthermore, enhanced inflammatory cell infiltration and mucus hypersecretion, thereby aggravating airway stenosis and remolding, as exhibited in the lung histopathology examination. PM2.5 exposure also elevated expressions of mucins such as MUC5ac and MUC5b and collagen-related biomarkers Collagen I and Collagen III in lung tissues of COPD rats. Additionally, the development of airway remolding and emphysema in COPD is highly linked to overwhelmed protease activity such as MMPs that participate in the degradation of elastic fibers and extracellular matrix [23]. We further observed an increment in MMPs (MMP-9 and 12) and decrement in their inhibitor TIMP-1 in COPD rats after PM2.5 exposure, which may result from excessive oxidants and inflammatory cells induced by PM2.5.

These results demonstrated that PM2.5 aggravates COPD through multiple ways, including oxidative stress, airway inflammation, mucus hypersecretion, proteases/antiprotease imbalance, and airway remodeling. We speculated that oxidative stress may play a central role. Once deposited on the surface of pulmonary bronchioli and alveoli, PM2.5 elicits oxidative stress, which further triggers a range of pathogenic processes starting with activation of proteases, then enhancing bronchial inflammation, goblet cell hyperplasia, and mucus hypersecretion, afterward increasing small airway fibrosis and collagen deposition, thereby resulting in elevated chronic inflammation and lung emphysema that demonstrate the development of COPD. However, further studies are required to clarify such hypothesis.

## 5. Conclusions

Altogether, our data suggest that PM2.5 exposure markedly increased oxidative stress, inflammation, proteases, hypersecretion of mucus and collagen, and airflow obstruction in rats with preexisting COPD. This study highlights the need to incorporate questions about exposure to pollution in patients who have COPD and importantly will allow new therapeutics to be evaluated in these models.

## Data Availability

The data used to support the findings of this study are available from the corresponding author upon request.

## Supplementary Materials

Supplementary Materials Figure S1: the daily atmospheric concentration of PM2.5 during the exposure period. Table S1: the distribution of particle size in the chamber. Table S2: the chemical composition analysis of PM2.5 samples.
