# Supplementary material for: Exposure to Air Pollution Exacerbates Inflammation in Rats with Preexisting COPD
Source: Mediators Inflamm. 2020 May 8;2020:4260204. doi: 10.1155/2020/4260204 (PMC7231193; doi:10.1155/2020/4260204)
Supplement: Supplementary Materials — Figure S1: the daily atmospheric concentration of PM2.5 during the exposure period. Table S1: the distribution of particle size in the chamber. Table S2: the chemical composition analysis of PM2.5 samples. [file 4260204.f1.docx]

**SUPPLEMENTARY MATERIALS**

**Exposure to air pollution exacerbates inflammation in rats with preexisting COPD**

Jing Wang^1,2^, Ya Li^1,2,3^, Peng Zhao^1,2^, Yange Tian^1,2^, Xuefang Liu^1,2^, Huihui He^1^, Rui Jia^1^, Brian G. Oliver ^1,2,4,5^, Jiansheng Li^1,2*^


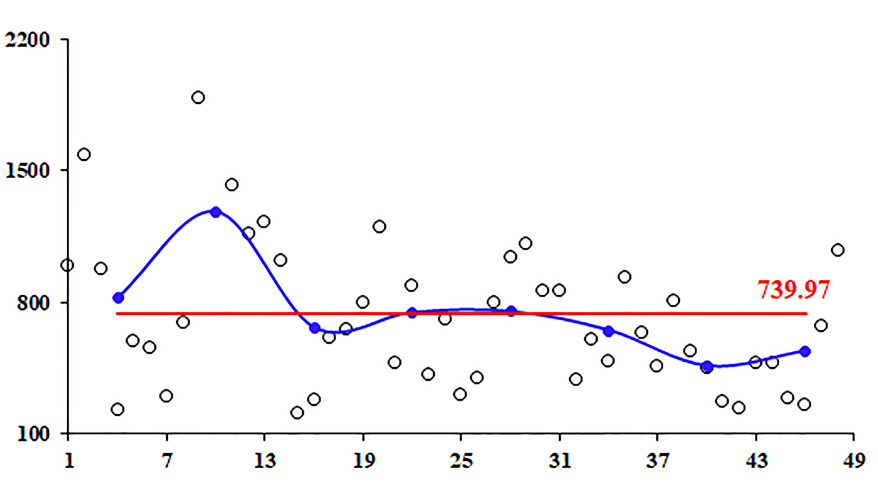


Figure S1: The mean concentration of PM2.5 in the exposure chamber from November 30th, 2018 to January 24th, 2019, in Zhengzhou, Henan province, China. The black dots showed the daily PM2.5 mean concentration of PM2.5. The maximum exposure that rats received was 2227.64 μg/m^3^, and the minimum exposure concentration was 205.12 μg/m^3^. The red line showed the mean daily concentration of PM2.5 (739.97 μg/m^3^) during the 8 weeks exposure, which was equivalent to 3~4-fold higher levels than ambient PM2.5. Moreover, the blue line showed the weekly mean concentration of PM2.5.

Table S1: The distribution of particle size in the chamber

|  | Number (#/cm^3^) | Surface (μm/cm^3^) | Mass (mg/cm^3^) |
| --- | --- | --- | --- |
| < 0.523 | 147.40 | 41.7 | 0.002 |
| 0.542 | 75.40 | 69.7 | 0.006 |
| 0.583 | 145.20 | 155.1 | 0.015 |
| 0.626 | 259.20 | 319.5 | 0.033 |
| 0.673 | 421.40 | 600.0 | 0.067 |
| 0.723 | 644.80 | 1060.0 | 0.128 |
| 0.777 | 767.40 | 1460.0 | 0.189 |
| 0.835 | 718.50 | 1580.0 | 0.219 |
| 0.898 | 575.30 | 1460.0 | 0.218 |
| 0.965 | 439.90 | 1290.0 | 0.207 |
| 1.037 | 330.30 | 1120.0 | 0.193 |
| 1.114 | 226.70 | 883.6 | 0.164 |
| 1.197 | 154.50 | 695.4 | 0.139 |
| 1.286 | 105.40 | 548.0 | 0.117 |
| 1.382 | 72.00 | 432.2 | 0.100 |
| 1.486 | 49.90 | 346.1 | 0.086 |
| 1.596 | 34.60 | 276.7 | 0.074 |
| 1.715 | 26.70 | 246.7 | 0.071 |
| 1.843 | 22.50 | 239.8 | 0.074 |
| 1.981 | 16.60 | 204.7 | 0.068 |
| 2.129 | 12.80 | 181.8 | 0.065 |
| 2.288 | 11.30 | 186.2 | 0.071 |
| 2.458 | 11.50 | 218.7 | 0.090 |
| 2.642 | 8.16 | 178.9 | 0.079 |
| 2.839 | 7.87 | 199.3 | 0.094 |
| 3.051 | 6.05 | 176.8 | 0.090 |
| 3.278 | 5.86 | 197.7 | 0.108 |
| 3.523 | 6.24 | 243.3 | 0.143 |
| 3.786 | 3.84 | 172.9 | 0.109 |
| 4.068 | 3.36 | 174.7 | 0.118 |
| 4.371 | 5.28 | 317.0 | 0.231 |
| 4.698 | 2.69 | 186.3 | 0.146 |
| 5.048 | 2.88 | 230.6 | 0.194 |
| 5.425 | 2.21 | 204.1 | 0.185 |
| 5.829 | 1.82 | 194.7 | 0.189 |
| 6.264 | 1.25 | 153.9 | 0.161 |
| 6.732 | 1.54 | 218.7 | 0.245 |
| 7.234 | 1.15 | 189.4 | 0.228 |
| 7.774 | 1.06 | 200.5 | 0.260 |
| 8.354 | 0.77 | 168.4 | 0.234 |
| 8.977 | 0.86 | 218.7 | 0.327 |
| 9.647 | 0.86 | 252.6 | 0.406 |
| 10.366 | 0.29 | 97.2 | 0.168 |
| 11.14 | 0.38 | 149.7 | 0.278 |
| 11.971 | 0.38 | 172.9 | 0.345 |
| 12.864 | 0.00 | 0.0 | 0.000 |
| 13.824 | 0.19 | 115.3 | 0.266 |
| 14.855 | 0.19 | 133.1 | 0.330 |
| 15.963 | 0.00 | 0.0 | 0.000 |
| 17.154 | 0.19 | 177.5 | 0.507 |
| 18.434 | 0.00 | 0.0 | 0.000 |
| 19.81 | 0.00 | 0.0 | 0.000 |
| Totals | 5330.00 | 18300.0 | 7.830 |

More than 90% particles were less than 2.5 μm.

Table S2 The chemical composition analysis of PM2.5 samples

|  |  | water-soluble ions (g/m^3^) | | |  | PAHs (μg/m^3^) | | | | | |  | microelements (g/m^3^) | | | | |
| --- | --- | --- | --- | --- | --- | --- | --- | --- | --- | --- | --- | --- | --- | --- | --- | --- | --- |
| component |  | NO_3_^-^ | SO_4_^2-^ | NH_4_^+^ | |  | NaP | BbF | BaP | PYR | FLT |  | Na | Pb | Al | K | Fe |
| concentration |  | 54.31 | 30.36 | 23.53 | |  | 38.22 | 58.42 | 44.53 | 25.78 | 37.18 |  | 1.24 | 2.42 | 1.73 | 2.39 | 4.21 |

PAH: polycyclic aromatic hydrocarbon, NaP: naphthalene, BbF: benzo[b]fluoranthene,

BaP: benzo[a]pyrene, PYR: [pyrene](javascript:;), FLT: fluoranthene.
